# Towards further harmonization of a glossary for exposure science—an ISES Europe statement

**DOI:** 10.1038/s41370-021-00390-w

**Published:** 2021-11-02

**Authors:** Gerhard Heinemeyer, Alison Connolly, Natalie von Goetz, Jos Bessems, Yuri Bruinen de Bruin, Marie A. Coggins, Peter Fantke, Karen S. Galea, Johannes Gerding, John D. Hader, Henri Heussen, Stylianos Kephalopoulos, Josephine McCourt, Paul T. J. Scheepers, Urs Schlueter, Martie van Tongeren, Susana Viegas, Maryam Zare Jeddi, Theo Vermeire

**Affiliations:** 1 German Federal Institute for Risk Assessment, retired, Berlin, Germany; 2 Centre for Climate and Air Pollution Studies, School of Physics and the Ryan Institute, National University of Ireland, University Road, H91 CF50 Galway, Ireland; 3 Swiss Federal Institute of Technology, Zurich, Switzerland; 4 Federal Office of Public Health, Bern, Switzerland; 5 Flemish Institute for Technological Research (VITO), Mol, Belgium; 6 European Commission, Joint Research Centre (JRC), Geel, Belgium; 7 Quantitative Sustainability Assessment, Department of Technology, Management and Economics, Technical University of Denmark, Kgs. Lyngby, Denmark; 8 Institute of Occupational Medicine (IOM), Edinburgh, EH14 4AP UK; 9 German Social Accident Insurance, Institution for the health and welfare services (BGW), Cologne, Germany; 10 Department of Environmental Science, Stockholm University, Stockholm, Sweden; 11 Cosanta BV, Schiphol-Oost, The Netherlands; 12 European Commission, Joint Research Centre (JRC), Ispra, Italy; 13 Radboud Institute for Health Sciences, Radboudumc, Nijmegen, The Netherlands; 14 Federal Institute for Occupational Safety and Health (BAuA), Dortmund, Germany; 15 Centre for Occupational and Environmental Health, School of Health Sciences, Faculty of Biology, Medicine and Health, University of Manchester, Manchester, UK; 16 NOVA National School of Public Health, Public Health Research Centre, Universidade NOVA de Lisboa, 1600–560 Lisbon, Portugal; 17 Comprehensive Health Research Center (CHRC), 1169–056 Lisbon, Portugal; 18 National Institute for Public Health and the Environment, Bilthoven, The Netherlands; 19 Present Address: European Chemical Industry Council (Cefic), Brussels, Belgium; 20 Present Address: European Commission, DG SANTE, Grange, Ireland

## Abstract

The use of aligned exposure science terminology is crucial for ease of comparison and appropriate interpretation of exposure information, regulatory reports, and scientific publications. Sometimes the use of different terminology in different contexts and areas of exposure science results in diverging interpretations of the same descriptor. During the development of the European strategy for exposure science, the need was identified to agree on a defined terminology requiring an evaluation of the commonly used terms, synonymous uses, and their relationships between each other. This paper presents the first steps in compiling the most important exposure-related terms from existing guidance documents and publications for exposure and risk assessment and adapting them to be useful for different contexts and areas. This initial step is intended to trigger discussion on terminology among exposure scientists around the globe and across regulatory and methodological silos. The glossary itself is intended as a living document to be hosted by the International Society for Exposure Science.

## Introduction

Using aligned and standardized terms in exposure science is crucial to facilitate harmonized interpretation of exposure information, unequivocal wording in publications and more straightforward comparison of data and results from exposure studies. This paper describes the outcome of a systematic review and analysis by the European Chapter of the International Society of Exposure Science (ISES Europe) of a core set of exposure-related terms. Several glossaries of terms that are used in exposure evaluations are published in guidance documents for exposure and risk assessments by national and international organizations [1–5]. However, these glossaries consider different terms and provide diverging definitions for varying contexts. In addition, some terms and their respective definitions evolved due to the increased relevance of exposure science in the regulatory field. Consequently, there is no comprehensive compilation available covering the most relevant and most frequently used terms in exposure science.

Historically, the first relevant terminology was developed by the International Programme on Chemical Safety (IPCS) of the World Health Organization (WHO) in a joint harmonization effort [4], based on a previously published framework of exposure terminology [6]. This was then taken up by the International Society for Exposure Analysis [7] and continuously updated by US-Environmental Protection Agency (EPA) as the glossary of the Exposure Factors Handbook [3].

Exposure evaluations are mostly prepared in the context of regulations, and therefore differences in regulatory requirements may result in the varying use and meaning of terms when applied to different regulatory settings. Furthermore, since regulation relates to specific countries, terminology may even differ in different parts of the world. This is also reflected by the different definitions used in scientific papers and studies, academic and industry data production, and education. Still, with exposure science evolving into a defined discipline, a common understanding of terminology across regulatory and scientific fields and across the globe becomes crucial. Therefore, even if this glossary is intended to support the ISES Europe Exposure Science strategy [8], it is important that a common terminology is adopted at an international level and the proposed update of the terms, therefore, relies mainly on international sources, with emphasis on European sources where definitions in international sources were lacking. Terminology is primarily based on an agreement of expert’s judgement laid down in the existing glossaries and also in the proposals made here by the ISES working group. Regarding the common understanding across exposure science fields, ISES Europe assembles exposure scientists from the different regulatory fields who reviewed the terminology and developed an understanding about key meanings and common contemporary use of specific terms [9, 10].

As a first step, a glossary containing the most frequently used terms is established as Supplemental Information to this paper. For constructing the glossary, the definitions used in different reference documents have been compared by expert judgement, and a definition was derived that was most suitable within the working group in terms of being the most (i) scientifically sound (ii) unambiguous, (iii) and broadly applicable in exposure science. The following steps will include discussion on an international level and expansion to integrate the variable usage of some terms into the definitions. This includes the evaluation of interlinks of the important terms of exposure, their synonyms, along with non-exhaustive lists of related abbreviations and different uses in different areas of exposure assessment across various regulatory contexts and possibly adjacent fields of science.

## Methods

Exposure experts within ISES Europe identified the most frequently used terms by 1. Identifying relevant glossaries published by leading international organizations, 2. listing the terms related to chemical exposure from the identified glossaries, and 3. evaluating the terms to identify the key, core terms to be included via expert judgement and discussions among the ISES Europe exposure scientists.

The relevant glossaries were taken from guidance documents published by international and European organizations, authorities and agencies (see Table 1). The glossaries from the European Chemicals Agency (ECHA) and the International Organization for Standardization (OECD) comprised different terminology documents, respectively. These are closely linked to the regulatory frameworks and exposure-related issues within their respective remits. For OECD, these guidance documents mostly refer to risk assessment and risk management at the workplace [11–14]. ISO/TS 21623 (2017) [15] includes a database of multiple terms and definitions that refers to different levels and combinations of terms. This database also refers to exposure terms other than those related to chemical stressors.

**Table 1:** Summary of glossaries published by organizations, authorities and agencies, and the number of terms identified related to chemical exposure.

Reference documents	Number of terms
1. The Glossary of terms in the US-EPA Exposure Factors Handbook, latest edition US-EPA [3]	208
2. The Description of selected key generic terms used in chemical hazard/risk assessment [1], identical to IPCS IPCS/OECD key generic terms (WHO/IPCS 2004, part 1) [4] used in chemical hazard/risk assessment. Joint Project with OECD on the Harmonization of Hazard/Risk Assessment Terminology	51
3. The WHO/IPCS Glossary of Key Exposure terminology (WHO/IPCS 2004, part 2) [4], identical to Zartarian et al. [7]	40
4. The ECHA guidance on information requirements and chemical safety assessment, Chapter R.20: Table of terms and abbreviations ECHA [2] and further ECHA guidance documents [19, 20]	26
5. The Scientific Opinion on Risk Assessment Terminology EFSA, published by the EFSA Scientific Committee EFSA [5]	14
6. The EFSA Glossary [21]	294
7. Terms from documents of the International Organisation for Standardization: ISO GUIDE 73 (2009) [11], 18158 (2016) [12], ISO/TS 21623 (2017) [13] and IEC 31010 (2019) [14], ISO-IEC 21623 (2017) [15]	See text below

Other glossaries published by OSHA [16] and IUPAC [17] were not taken into consideration because they are primarily focused on hazard assessment and toxicology and thus do not have a major influence on exposure assessment terminology.

In the case that an important term had not been defined in one of these official glossaries, definitions proposed in other publications have been taken into account (e.g., for the term “exposome”). If no reference at all was available, a definition was provided by the involved experts and the field “defined in glossary” is blank.

The proposed glossary of exposure terms includes the terms, the major synonyms, and whether they are defined in the glossaries of the above-mentioned guidance documents. Whenever the text was used verbatim (or in some instances with slight edits), the reference is given after the definition. Otherwise, the definition has been discussed and amended or developed to be more applicable in an exposure science setting and agreed upon by the exposure experts in ISES Europe who are authoring this paper and is proposed by ISES Europe as a new definition.

In summary, the method to establish the harmonized terminology involved the collection of appropriate glossaries/guidance documents, the evaluation of previously/currently used terminology and exposure science expert working groups reviewed and determined the final glossary for the exposure science field. The approach leading to the glossary of recommended exposure-related terms was guided by the following decision rules:

1. Glossaries/guidance documents used are peer-evaluated.
2. Glossaries/guidance documents used are from organizations authoritative on exposure and risk assessment.
3. Glossaries/guidance documents used are applied at an international level.
4. If terms are defined in more than one authoritative document, preference is given to definitions harmonized at an international level (mostly appearing in the WHO/IPCS glossary or updates in the US-EPA glossary) and referenced as such.
5. In the case that an important term has not been defined in one of the authoritative documents, definitions proposed in other scientific publications are taken into account or were defined by ISES Europe exposure scientists and through working group discussions.
6. Each definition is subject to review by ISES Europe exposure scientists and, if the proposed definitions are considered unsatisfactory or incomplete, the definition is adapted and appears in the glossary as an ISES Europe proposal.

## Results

A total of 49 key terms were identified. These are listed alphabetically, along with details of the reference documents in which they were defined, any common synonyms and ISES Europe’s proposed definition. They are listed in the “Supplemental Information”

An evaluation of the terms listed in the glossaries reveals that only the term “*exposure scenario*” is listed in each glossary. The terms “*exposure*” and “*exposure scenario”* are listed in all documents mentioned in Table 2, and “e*xposure assessment*” and “*dose*” in four of them (Table 2). The term “uncertainty” is listed in five documents and is broadly discussed in a separate guidance document published by WHO/IPCS [18]. All other terms are listed three times or less. There are 228 terms that are listed only once. This evaluation demonstrates that currently, there are only some very general terms exist that can be regarded as “harmonized”.

**Table 2:** Occurrence of terms listed in more than three terminology guidance documents (indicated by “x”).

	WHO/IPCS (part 2) [4]; Zartarian et al. [7]	US-EPA [3]	OECD [1]; WHO/IPCS (part 1) [4]	ECHA [2, 19, 20]	EFSA [5, 21]	ISO documents [11–15]
Exposure scenario	x	x	x	x	x	x
Exposure	x	x	x		x	x
Dose	x	x	x			x
Exposure assessment	x	x		x		x
Uncertainty	x	x	x		x	x

## Discussion and conclusions

The need for harmonized terminology for exposure science was demonstrated by different definitions identified among different reviewed documents, including ISO’s and regulatory agency guidance documents. Previous terminology documents had varying scopes and objectives (e.g., food safety, chemicals safety), which in some instances narrowed the perspective. However, there are a number of exposure-related terms that can be aligned across different fields of exposure science. In order to promote common understanding between those different fields, this document attempted to align the terminology as far as possible by relying on the multitude of professional backgrounds represented in ISES Europe. To support the identity of exposure science, the use of these terms in publications will be encouraged by ISES Europe and used throughout the papers related to the European Strategy for Exposure Science.

The comparison and update of exposure-related terms and their definitions show the need for a thorough re-evaluation of the vocabulary and consistent and harmonized use in exposure science, within and across regulations. However, differences in the understanding and meanings in particular regulations on regional, national and international levels, as well as links between the terms and abbreviations, have not been considered in this document. Many definitions mentioned in guidance documents are conventions from stakeholder discussions with regard to regulatory issues. However, a proper scientific evaluation of the uses of the terms, their synonyms, and their relationships within the different fields of exposure assessment has not been done. Terms used similarly in different regulations might have diverging and different meanings, which should be documented and analyzed. This should include a comparison of terms used in regulatory exposure assessments and other areas of exposure science. Therefore, as a second step and long-term goal of ISES Europe is to develop a standard approach that integrates the different understandings of exposure terms and their dependencies.

The contribution of exposure assessment in human and environmental risk assessment is essential and needs a globally adopted terminology. Also, in this respect, the proposed glossary is meant as an update of existing definitions. As such, it is the starting point for a more international discussion on terminology that has already started within the International Society on Exposure Science and ideally would trigger an update of the exposure terminology at the OECD or WHO level.

The presented short core list of exposure-related terms should be understood as a preliminary glossary of key terms, which will be developed into a living document to be enriched with further terminology and evaluated with more international experts with the ultimate objective of achieving a more complete, thoroughly evaluated compilation that goes beyond this glossary.

## Disclaimer

The views and opinions expressed in this article are those of the authors and do not necessarily reflect the official policy or position of any agency, organization, employer or company.

## Supplementary Information

Supplementary Information
