# Supplementary material for: Towards further harmonization of a glossary for exposure science—an ISES Europe statement
Source: J Expo Sci Environ Epidemiol. 2021 Nov 2;32(4):526–9. doi: 10.1038/s41370-021-00390-w (PMC9349032; doi:10.1038/s41370-021-00390-w)
Supplement: Supplementary file 1 — Supplementary Information [file 41370_2021_390_MOESM1_ESM.docx]

**Supplementary Information**

**Towards further harmonization of a glossary for exposure science – an ISES Europe statement**

Gerhard Heinemeyer^1^*, Alison Connolly^2^, Natalie von Goetz^3,4^*, Jos Bessems^5^, Yuri Bruinen de Bruin^6†^, Marie A. Coggins^2^, Peter Fantke^7^, Karen S. Galea^8^, Johannes Gerding^9^, John D. Hader^10^, Henri Heussen^11^, Stylianos Kephalopoulos^12^, Josephine McCourt^6††^, Paul T.J. Scheepers^13^, Urs Schlueter^14^, Martie van Tongeren^15^, Susana Viegas^16,17^, Maryam Zare Jeddi^18^, Theo Vermeire^18^

| ^1^ German Federal Institute for Risk Assessment, Berlin, Germany, retired, gerhardheinemeyer@o2mail.de  *Corresponding author |
| --- |
| ^2^ Centre for Climate and Air Pollution Studies, School of Physics and the Ryan Institute, National University of Ireland, University Road, Galway, Ireland, H91 CF50  ^3^ Swiss Federal Institute of Technology, Zurich, Switzerland, Natalie.von.goetz@chem.ethz.ch  ^4^ Federal Office of Public Health, Bern, Switzerland |
| *Corresponding author  ^5^ Flemish Institute for Technological Research (VITO), Mol, Belgium |
| ^6^ European Commission, Joint Research Centre (JRC), Geel, Belgium  † Current affiliation: European Chemical Industry Council (Cefic), Brussels, Belgium  †† Current affiliation: European Commission, DG SANTE, Grange, Ireland |
| ^7^ Quantitative Sustainability Assessment, Department of Technology, Management and Economics, Technical University of Denmark, Kgs. Lyngby, Denmark |
| ^8^ Institute of Occupational Medicine (IOM), Edinburgh, EH14 4AP, UK |
| ^9^ German Social Accident Insurance, Institution for the health and welfare services (BGW), Cologne, Germany |
| ^10^ Department of Environmental Science, Stockholm University, Sweden |
| ^11^ Cosanta BV, Schiphol-Oost, The Netherlands  ^12^ European Commission, Joint Research Centre (JRC), Ispra, Italy |
| ^13^ Radboud Institute for Health Sciences, Radboudumc, Nijmegen, Netherlands |
| ^14^ Federal Institute for Occupational Safety and Health (BAuA), Dortmund, Germany |
| ^15^ Centre for Occupational and Environmental Health, School of Health Sciences, Faculty of Biology, Medicine and Health, University of Manchester, Manchester, United Kingdom |
| ^16^ NOVA National School of Public Health, Public Health Research Centre, Universidade NOVA de Lisboa, 1600–560 Lisbon, Portugal  ^17^ Comprehensive Health Research Center (CHRC), 1169–056 Lisbon, Portugal |
| ^18^ National Institute for Public Health and the Environment, Netherlands |
|  |

**Preamble**

The proposed glossary is intended to be broadly applicable in different areas of exposure science. The definitions given in the referenced documents were taken verbatim, as cited. If adaptations were necessary, the citation was mentioned with "based on", and the respective change explained.

If the ISES Europe proposal is entirely based on a definition from an authoritative organization, it is referenced as such. In the case that an important term had not been defined in one of these official glossaries, definitions proposed in other publications have been taken into account (e.g. for the term “exposome”). If no reference at all was available, a definition was provided by the involved experts and the field “defined in glossary” is blank.

Proposals without a reference are definitions formulated and agreed upon by ISES Europe exposure scientists.

**List /Table of terms**

| ***Term:*** | Absorption barrier |
| --- | --- |
| ***Defined in glossary:*** | US-EPA (2011) |
| **ISES-Europe proposal:** | Any exposure surface that may retard the rate of penetration of an agent into a target. Examples include the skin, respiratory tract lining, and gastrointestinal tract wall (US-EPA, 2011) |
| ***Term:*** | Activity pattern data |
| ***Defined in glossary:*** | US-EPA (2011) WHO/IPCS (2004, part 2) |
| **ISES-Europe proposal:** | Data that characterize the habits/behaviour of the target population (WHO/IPCS, 2004, part 2). |
| ***Term:*** | Activity pattern |
|  |  |
| ***ISES-Europe proposal:*** | Habits/behaviour of the target population.^[[1]](#footnote-1)^ |
|  |  |
| ***Term:*** | Acute exposure |
| ***Synonymous terms:*** | Short-term exposure |
| ***ISES-Europe proposal:*** | See: Short-term exposure |
|  |  |
|  |  |
| ***Term:*** | Agent |
| ***Synonymous terms:*** | includes the terms substance, chemical, biological agent, physical agent, Stressor |
| ***Defined in glossary:*** | WHO/IPCS (2004; part 2), ISO/TS 21623 (2017) |
| ***ISES-Europe proposal:*** | A chemical, biological, or physical entity that contacts a target (WHO/IPCS 2004, part 2). |
|  |  |
| ***Term:*** | Aggregate (d) exposure |
| ***Defined in glossary:*** | US-EPA (2011), ECHA (2016) |
| ***ISES-Europe proposal:*** | Exposure to one agent from different exposure sources, via different exposure pathways and/or exposure routes. |
| ***Remark:*** | If all routes and sources have been considered, the aggregate exposure is equivalent to "total exposure" to a specific agent or stressor. |
|  |  |
| ***Term:*** | Background level |
| ***Defined in glossary:*** | WHO/IPCS (2004, part 2) |
| ***ISES-Europe proposal:*** | The amount of an agent in a medium (e.g., air, water, soil, biological media) that is not attributed to the source(s) under investigation in an exposure assessment. Background level(s) can be naturally occurring and/or the result of human activities. (WHO/IPCS, 2004, part 2). |
| ***Remark:*** | Natural background is the concentration of an agent in a medium that occurs naturally or is not the result of human activities. |
|  |  |
| ***Term:*** | Bioaccumulation |
| ***Defined in Glossary:*** | ISO/TS 21623 (2017) |
| ***ISES-Europe proposal:*** | The increase in internal concentration of an agent in an organism due to repeated exposure and due to all routes of exposure where metabolism and excretion have not been able to cope with the repeated exposure (and absorption) over time (new definition, based on van Leeuwen & Vermeire, 2007). |
| ***Remark:*** | Bioconcentration often refers to the net result of uptake, distribution and elimination of a substance due to water-borne exposure of an organism. Together with bioaccumulation is may lead to biomagnification by which tissue concentrations of bioaccumulated chemicals increase as the chemical passes up to two or more trophic levels (van Leeuwen & Vermeire, 2007). |
|  |  |
| ***Term:*** | Bioavailability |
| ***Synonymous terms:*** | Systemic availability |
| ***Defined in glossary:*** | US-EPA (2011), WHO/IPCS (2004, part 2), ISO/TS 21623 (2017) |
| ***ISES-Europe proposal:*** | The rate and extent to which an agent is systemically absorbed by an organism via all possible exposure routes and is available for metabolism or interaction with biologically significant receptors. Bioavailability involves both release from a medium (if present) and absorption by an organism. |
|  |  |
| ***Term:*** | Biomarker of exposure |
| ***Synonymous terms:*** | Biological marker of exposure |
| ***Defined in glossary:*** | US-EPA (2011), WHO/IPCS (2004, part 2), EFSA (2017), ISO/TS 21623 (2017). |
| ***ISES-Europe proposal:*** | An indicator of the exposure of biological systems to an agent. Biomarkers of exposure refer to chemical-analytical measurements of the analyte in the biological system. |
|  |  |
|  |  |
| ***Term:*** | Biomonitoring |
| ***Defined in glossary:*** | EFSA (2017), IUPAC (2007) |
| ***ISES-Europe proposal:*** | The measurement of chemical agents and/or their metabolites, or markers of subsequent health effects, in organisms and biological media such as tissues, cells or fluids (based on HBM4EU 2020 leaflet). |
|  |  |
| ***Term:*** | Chronic exposure |
| ***Synonymous terms*** | Long-term exposure |
| ***ISES-Europe proposal:*** | See: Long-term exposure |
|  |  |
| ***Term:*** | Cumulative exposure |
| ***Synonymous terms:*** | Combined exposure, mixture exposure |
| ***Defined in glossary:*** | US EPA (2002) |
| ***ISES-Europe proposal:*** | [Total](#total_exposure) or [aggregate exposure](#Aggregated_exposure)s (or doses) for multiple chemicals or stressors evaluated together. |
| ***Remark:*** | Broadly discussed in WHO/IPCS (2009) and Meek et al. (2011). Cumulated exposure may vary from a small number to mixtures of a broad variety of agents.  Cumulative exposure is generally understood for substances having similar actions.  Within (occupational) epidemiology, cumulative exposure is generally and frequently to describe chronic exposure (exposure level > year) |
|  |  |
| ***Term:*** | Dose |
| ***Defined in glossary:*** | US-EPA (2011) WHO/IPCS (2004, part 2) OECD (2003**)** EFSA (2017), ISO/TS 21623 (2017) |
| ***ISES-Europe proposal:*** | The amount of an agent that enters a target due to total exposure over a certain time-period. Dose can refer to the amount of chemical taken in by the entire target organism or the amount taken in by specific target cells within the target organism. In the case of absorption, the dose is an absorbed dose/uptake dose (see uptake); otherwise, it is an intake dose. (Adopted from US-EPA, 2011; WHO-IPCS, 2004 part 2,). |
|  |  |
| ***Term:*** | Emission |
| ***Defined in glossary*** | ISO/TS 21623 (2017) |
| ***Synonymous terms:*** | Release |
| ***ISES-Europe proposal:*** | Release of an agent from an exposure source, including discharges into the wider environment (van Leeuwen and Vermeire, 2007). |
|  |  |
| ***Term:*** | Exposome |
| ***ISES-Europe proposal:*** | The totality of a person's exposure from conception to death (based on Wild, 2005). |
| ***Remark:*** | This term is not mentioned in any of the reference documents mentioned previously. However, it is an emerging, important term that should be considered in a list on exposure terminology. The proposed definition is a condensation of wording in Wild, 2005 ("At its most complete, the exposome encompasses life-course environmental exposures (including lifestyle factors), from the prenatal period onwards.") |
|  |  |
| ***Term:*** | Exposure |
| ***Defined in glossary:*** | US-EPA (2011), WHO/IPCS (2004, part 2), OECD (2003), EFSA (2017), ISO/TS 21623 (2017) |
| ***ISES-Europe proposal:*** | Contact between an agent and a target. Exposure is measured as the concentration or amount of a particular agent that reaches a target organism, system or (sub) population in a specific exposure frequency for a defined exposure duration (OECD, 2003; WHO/IPCS, 2004 part 1). |
| ***Remark:*** | In WHO/IPCS (2008), exposure is characterized by the three elements "exposure scenario", "exposure model", and "exposure parameters". |
|  |  |
| ***Term:*** | Exposure assessment |
| ***Defined in glossary:*** | OECD, 2003; WHO/IPCS, 2004 part 1; US-EPA, 2011 WHO/IPCS, 2004 part 2; ECHA (2013, 2016, 2016a), EFSA (2012, 2017), ISO/TS 21623 (2017) |
| ***ISES-Europe proposal:*** | The process of estimating or measuring the magnitude, frequency, and duration of exposure to an agent, along with the number and characteristics of the population exposed. (US-EPA, 2011). |
|  |  |
| ***Term:*** | Exposure duration |
| ***Defined in glossary:*** | US-EPA (2011), WHO/IPCS (2004, part 2), ISO/TS 21623 (2017) |
| ***ISES-Europe proposal:*** | The length of time over which continuous or intermittent contacts occur between an agent and a target.# (WHO/IPCS, 2004 part 2). |
| ***Remar*** | For example, if an individual is in contact with an agent for 10 min, it is short term exposure.  If the contact is every day for 300 days over a 1-year time period, the exposure duration is 1 year; it is long-term exposure |
|  |  |
| ***Term:*** | Exposure event |
| ***Defined in glossary:*** | US-EPA (2011), WHO/IPCS (2004, part 2), ISO/TS 21623 (2017) |
| ***ISES-Europe proposal:*** | A single event during which there is continuous contact between an agent and a target. The event is normally of a short duration (less than 24 hours). |
| ***Remark:*** | The attribute "continuous contact" in this context means that there is no interruption of the exposure. |
|  |  |
| ***Term:*** | Exposure factor |
| ***Defined in glossary:*** | US-EPA (2011), ISO/TS 21623 (2017) |
| ***ISES-Europe proposal:*** | Exposure factors are exposure parameters related to human behaviour and characteristics that help determine an individual's exposure to an agent (US-EPA, 2011). |
|  |  |
| ***Term:*** | Exposure frequency |
| ***Defined in glossary:*** | US-EPA (2011), WHO/IPCS (2004, part 2) |
| ***ISES-Europe proposal:*** | The number of exposure events in an exposure duration (US-EPA; 2011; WHO/IPCS 2004 part 2). |
|  |  |
| ***Term:*** | Exposure model |
| ***Defined in glossary:*** | WHO/IPCS (2004, part 2) |
| ***ISES-Europe proposal:*** | A conceptual or mathematical representation of one or more exposure processes. WHO/IPCS (2004, part 2). |
|  |  |
| ***Term:*** | Exposure parameter |
| ***ISES-Europe proposal:*** | All (model) data that are necessary to calculate exposure, including exposure factor and agent concentrations. |
| ***Remark:*** | In WHO/IPCS (2008), the term "exposure parameter" is mentioned as one of the three elements of exposure, namely exposure scenario, exposure model and exposure parameter.  The ISO/TS 21623 (2017) term refers to temperature differences and cannot be applied for exposure assessment. |
|  |  |
| ***Term:*** | Exposure pathway |
| ***Defined in glossary:*** | US-EPA (2011), WHO/IPCS (2004, part 2), ISO/TS 21623 (2017) |
| ***ISES-Europe proposal:*** | The course an agent takes from the exposure source to the target (WHO/IPCS 2004, part 2). |
|  |  |
| ***Term:*** | Exposure route |
| ***Synonymous terms:*** | Route of exposure |
| ***Defined in glossary:*** | US-EPA (2011) WHO/IPCS (2004, part 2), ISO/TS 21623 (2017) |
| ***ISES-Europe proposal:*** | The means through which an agent enters a target organism after contact (e.g. by ingestion, inhalation, or dermal absorption) (US-EPA 2011), WHO/IPCS 2004, part 2). |
|  |  |
| ***Term:*** | Exposure Scenario |
| ***Defined in glossary:*** | US-EPA (2011), WHO/IPCS (2004, part 2), OECD (2003), ECHA (2013, 2016, 2016a), ISO/TS 21623 (2017) |
| ***ISES-Europe proposal:*** | A combination of facts, assumptions, and inferences that define a discrete situation where potential exposures may occur. These may include the exposure source, the exposed target population, and the time frame of exposure, microenvironment, and activities. Scenarios are often created to aid exposure assessors in estimating exposure (WHO/IPCS, 2004). |
| ***Remark:*** | **I**n regulatory risk assessments, a range of exposure scenarios is often investigated (e.g. realistic as well as worst-case scenarios, meaning an average as well as an extreme exposure situation).  In the European Chemicals Regulation (REACH), the Exposure scenario is defined by regulation (European Union, 2006). |
|  |  |
| ***Term:*** | Exposure Science |
| ***Defined in glossary*** | No definition available |
| ***ISES-Europe proposal:*** | Exposure science studies the contact between stressors and receptors and the associated exposure sources, exposure pathways and processes potentially leading to impacts on human health and the natural and built environment. Stressors primarily refer to chemical, biological, and physical agents, and receptors range from molecules, cells, and organs to humans and other organisms. (based om Fandtke et al., 8) |
|  |  |
| ***Term:*** | Exposure Source |
| ***Synonymous terms:*** | Source of exposure |
| ***Defined in glossary:*** | US-EPA (2011), WHO/IPCS (2004, part 2), ISO/TS 21623 (2017) |
| ***ISES-Europe proposal:*** | The origin of an agent entering an [exposure pathway](#exposure_pathway) for the purposes of an exposure assessment (based on WHO/IPCS; 2004, part2). |
|  |  |
| ***Term:*** | External exposure |
| ***Synonymous terms:*** | external dose |
| ***ISES-Europe proposal:*** | Exposure of an organism at an exposure barrier before it is taken up by an organism. |
|  |  |
| ***Term:*** | Internal dose |
| ***Synonymous terms:*** | Absorbed dose |
| ***Defined in glossary:*** | US-EPA (2011) |
| ***ISES-Europe proposal:*** | The amount of an agent that enters the organ system by crossing an absorption barrier (US-EPA, 2011). |
|  |  |
| ***Term:*** | Intake |
| ***Defined in glossary:*** | US-EPA (2011) WHO/IPCS (2004, part 2) |
| ***ISES-Europe proposal:*** | The process by which a substance crosses the outer boundary of an organism without passing an absorption barrier (e.g., through ingestion or inhalation) (US-EPA, 2011). |
|  |  |
| ***Term:*** | Intake fraction |
| ***ISES-Europe proposal:*** | Fraction of chemical mass emitted into the environment that eventually passes into an individual or a population through intake (i.e., inhalation or ingestion) or dermal absorption. (based on Bennet et al., 2002) |
|  |  |
| ***Term:*** | Long-term exposure |
| ***Synonymous terms*** | Chronic exposure |
| ***Defined in glossary:*** | US-EPA (2011), WHO/IPCS (2004, part 2, EFSA (2017) |
| ***ISES-Europe proposal:*** | Continuous exposure, or multiple exposures, occurring over an extended period of time or a significant fraction of the organism's lifetime (US-EPA 2011). |
| ***Remark*** | In human toxicology, chronic exposure corresponds to chronic toxicity, which is defined as the effects observed following repeated exposure by the oral, dermal, or inhalation route for more than approximately 10% of the life span in humans (more than approximately 90 days to 2 years in typically used laboratory animal species) and in environmental toxicology as the effects observed following multiple exposures occurring over an extended period of time or over a significant fraction of an organism's lifetime. Chronic exposures can be understood as a special case of "long-term exposure". |
|  |  |
| ***Term:*** | Monitoring |
| ***Defined in glossary*** | ISO/TS 21623 (2017) |
| ***ISES-Europe proposal:*** | Measuring one or more agents/stressors in environmental media or in body fluids or tissues (derived from the definition of biomonitoring). |
| ***Remark:*** | Many different types of monitoring exist: risk monitoring, biomonitoring, exposure monitoring, and human biomonitoring, environmental monitoring, monitoring at the workplace (e.g. personal air sampling). Definitions exist in terms of the particular types of monitoring and should be evaluated. |
|  |  |
| ***Term:*** | Reference Dose (RfD) |
| ***Defined in glossary:*** | US-EPA (2011), WHO/IPCS (2004, part 1), ISO/TS 21623 (2017) |
| ***ISES-Europe proposal:*** | An estimate of the daily dose of an agent for a human population that is likely to be without deleterious effect even if continued exposure occurs over a lifetime (OECD, 2003).  In the case of inhalation exposure, the reference "dose" is a reference concentration. |
|  |  |
| ***Term:*** | Risk assessment |
| ***Defined in glossary:*** | US-EPA (2011), WHO/IPCS (2004, part 1), EFSA (2017)), ISO/TS 21623 (2017) |
| ***ISES-Europe proposal:*** | A process intended to calculate or estimate the risk for a specified target organism, system, or (sub)population, including the identification of related uncertainties, following exposure to a particular agent, taking into account the inherent characteristics of the agent of concern as well as the characteristics of the specific target system. The risk assessment process includes the following steps: hazard identification, problem formulation), hazard characterization (related term: Dose–response assessment), exposure assessment, and risk characterization. Risk assessment is the first component in a risk analysis process ((US-EPA, 2011). |
| ***Remark:*** | Risk assessment may also include the assessment of statistical correlations between an analyte level (exposure biomarker) and an effect level (effect biomarker or apical effect). |
|  |  |
| ***Term:*** | Risk characterization |
| ***Defined in glossary:*** | US-EPA (2011), WHO/IPCS (2004, part 2), EFSA (2017), ISO/TS 21623 (2017) |
| ***ISES-Europe proposal:*** | The qualitative and, wherever possible, quantitative determination, including related uncertainties, of the probability of occurrence of known and potential adverse effects of an agent in a given organism, system, or (sub) population, under defined exposure conditions.  Risk characterization is the fourth step in the risk assessment process (WHO/IPCS, 2004) |
|  |  |
| ***Term:*** | Risk management |
| ***Defined in glossary:*** | WHO/IPCS (2004, part 1), EFSA (2017), ISO/TS 21623 (2017) |
| ***ISES-Europe proposal:*** | Decision-making process involving considerations of political, social, economic, and technical factors with relevant risk assessment information relating to a hazard so as to develop, analyze, and compare regulatory and non-regulatory options and to select and implement appropriate response option(s) to that hazard.  Risk management comprises three elements: risk evaluation; emission and exposure control; and risk monitoring (OECD, 2003). |
| ***Remark:*** | Risk management is primarily supported by exposure assessment, which delivers the essential information needed for risk reduction. |
|  |  |
| ***Term:*** | Sensitivity analysis |
| ***Defined in glossary:*** | US-EPA (2011), ISO/TS 21623 (2017) |
| ***ISES-Europe proposal:*** | Process of changing one variable of a quantitative analysis while keeping the others constant to determine its effect on the output. Generally, this procedure varies each uncertain quantity between its credible lower and upper bounds (holding all others at their nominal values, such as medians) and computes the results of each combination of values. The results help to identify the variables that have the greatest effect on exposure estimates and help focus further information-gathering efforts (US-EPA, 2011) |
|  |  |
| ***Term:*** | Short-term exposure |
| ***Synonymous terms*** | Acute exposure |
| ***Defined in glossary:*** | US-EPA (2011), WHO/IPCS (2004, part 2), ISO/TS 21623 (2017) |
| ***ISES-Europe proposal:*** | A single exposure to a toxic agent/stressor which may result in severe biological harm or death. Short-term exposures are usually characterized as lasting no longer than a day, as compared to longer, continuing exposure over a period of time (according to the definition of 'acute exposure' in US-EPA, 2011). |
| ***Remark:*** | Acute exposure corresponds to the acute (severe) effects observed following a single exposure or an exposure of a duration of less than a day. In ecotoxicology, the term short-term toxicity or acute toxicity is used for the effects observed after an exposure that is short in relation to the lifespan of an organism. Acute exposure can be understood as a special case of "Short-term exposure." |
| ***Term:*** | Stressor |
| ***Defined in glossary:*** | WHO/IPCS (2004, part 2), EFSA (2017) |
| ***ISES-Europe proposal:*** | Any entity, stimulus, or condition that can modulate normal functions of an organism or ecosystem or induce an adverse response (e.g., an agent, lack of food, drought) (WHO/IPCS, 2004, part 2). |
| ***Remark:*** | Stressors primarily refer to adverse effects from chemical, biological, and physical agents, which induce the adverse effects, and receptors range from molecules, cells, and organs to humans and other organisms and ecosystems (Fantke et al., 2020). |
|  |  |
| ***Term:*** | Target |
| ***Defined in glossary:*** | US-EPA (2011), WHO/IPCS (2004, part 2), ISO/TS 21623 (2017) |
| ***ISES-Europe proposal:*** | Any biological entity that receives an exposure or a dose (e.g., a human, a human population, or a human organ, cell or receptor) (WHO/IPCS 2004, part 2) |
|  |  |
| ***Term:*** | Target Population |
| ***Defined in glossary:*** | EFSA (2017) |
| ***ISES-Europe proposal:*** | Group or subgroup of human or environmental populations defined by a particular physiological status (species, age-range and gender, disease status). Because of their particular physiological status, pregnant and lactating women are specific target populations. |
|  |  |
| ***Term:*** | Total exposure |
| ***Defined in glossary***  ***ISES-Europe proposal:*** | US-EPA (2011  The sum of exposure originating from all exposure sources in any consumer products, workplace exposures and via the environment to a particular agent.  (in accordance with the US-EPA (2011) definition of total food exposure). |
| ***Remark:*** | Total exposure may refer to the total of "Aggregate (d) exposure" or of “Cumulative exposure ”. |
|  |  |
| ***Term:*** | Toxicokinetics |
| ***Synonymous terms:*** | Pharmacokinetics |
| ***Defined in glossary:*** | US-EPA (2011), EFSA (2017), ISO/TS 21623 (2017) |
| ***ISES-Europe proposal:*** | The entry into, the distribution through and the metabolism and excretion of an agent through an organism as a function of dose and time. |
|  |  |
| ***Term:*** | Uptake |
| ***Synonymous terms:*** | Absorption |
| ***Defined in glossary:*** | US-EPA (2011)), ISO/TS 21623 (2017) |
| ***ISES-Europe proposal:*** | The process by which an agent crosses an absorption barrier and is absorbed into the body (US-EPA, 2011). |
|  |  |
| ***Term:*** | Uncertainty |
| ***Defined in glossary:*** | US-EPA (2011), WHO/IPCS (2004, part 1), EFSA (2017), ISO/TS 21623 (2017) |
| ***ISES-Europe proposal:*** | Lack of knowledge that can lead to inaccurate or biased estimates of exposure. Can refer to exposure scenario, exposure parameter, and exposure model. |
|  |  |
| ***Term:*** | Variability |
| ***Defined in glossary:*** | US-EPA (2011), EFSA (2017), ISO/TS 21623 (2017) |
| ***ISES-Europe proposal:*** | True heterogeneity across locations, time, or individuals in a human or environmental population. Variability can affect the precision and accuracy of exposure estimates and the degree to which they can be generalized. |
|  |  |

**References**

1. Bennett DH, Margni MD, McKone TE, Jolliet O (2002) Intake fraction for multimedia pollutants: a tool for life cycle analysis and comparative risk assessment. Risk Anal. 2002 (5):905-18.
2. ECHA (2013) Guidance on information requirements and chemical safety assessment. Chapter R.20: Table of terms and abbreviations.
   ECHA-12-G-26.1-EN, European Chemicals Agency, <http://echa.europa.eu/>
3. ECHA (2016) Guidance on Information Requirements and Chemical Safety Assessment Chapter R.15: Consumer exposure assessment. Version 3.0 - July 2016. ECHA-16-G-07-EN. European Chemicals Agency, <http://echa.europa.eu/>
4. ECHA (2016a) Guidance on Information Requirements and Chemical Safety Assessment. Part D: Framework for exposure assessment. Version 2.0. Reference: ECHA-16-G-08-EN. Cat. Number: ED-01-16-447-EN-N ISBN: 978-92-9495-080-2, DOI: 10.2823/479399 <https://echa.europa.eu/documents/10162/13632/information_requirements_r15_en.pdf>
5. EFSA (2012). EFSA Scientific Committee; Scientific Opinion on Risk Assessment Terminology. Parma2, Italy: European Food Safety Authority. The EFSA Journal, No. 2664, Vol.. 10(5). <https://doi.org/10.2903/j.efsa.2012.2664>
6. EFSA (2017) Glossary. https://www.efsa.europa.eu/en/glossary-taxonomy-terms.
7. European Union (2006) REGULATION (EC) No 1907/2006 OF THE EUROPEAN PARLIAMENT AND OF THE COUNCIL of 18 December 2006 concerning the Registration, Evaluation, Authorization and Restriction of Chemicals (REACH), establishing a European Chemicals Agency, amending Directive 1999/45/EC and repealing Council Regulation (EEC) No 793/93 and Commission Regulation (EC) No 1488/94 as well as Council Directive 76/769/EEC and Commission Directives 91/155/EEC, 93/67/EEC, 93/105/EC and 2000/21/EChttp://data.europa.eu/eli/reg/2006/1907/oj/eng, CHAPTER 2, Definitions and general provision, Article 3, Definitions, paragraph 37
   <https://eur-lex.europa.eu/legal-content/EN/TXT/PDF/?uri=CELEX:02006R1907-20190702&from=en>
8. Fantke P, von Goetz N, Schlüter U, Bessems J, Connolly A, Dudzina T, Ahrens A, Bridges J, Coggins MA., Conrad A, Hänninen O, Heinemeyer G, Kephalopoulos S, McLachlan M, Meijster T, Poulsen V, Rother D, Vermeire T, Viegas S, Vlaanderen J, Zare Jeddi M, Bruinen de Bruin Y (2020). Building a European exposure science strategy. Journal of Exposure Science & Environmental Epidemiology, <https://doi.org/10.1038/s41370-019-0193-7>
9. HBM4EU (2020) Human Biomonitoring for Europe. <https://www.hbm4eu.eu/about-hbm4eu/> (last access: 17 Jan 2020)
10. ISO/TS 21623 (2017) Workplace exposure — Assessment of dermal exposure to nano-objects and their aggregates and agglomerates (NOAA). <https://www.iso.org/obp/ui#iso:std:iso:ts:21623:ed-1:v1:en>
11. IUPAC (2007) Glossary of terms used in toxicology. IUPAC recommendations 2007. International Union of Pure and Applied Chemistry. Chemistry and Human Health Division. 2nd EDITION. Pure Appl. Chem., Vol. 79, No. 7, pp. 1153–1344, 2007. doi:10.1351/pac200779071153
12. Meek ME, Boobis AR, Crofton KE, Heinemeyer G, Van Raaij M, Vickers C (2011) Risk assessment of combined exposure to multiple chemicals: a WHO/IPCS framework. Regul Toxicol Pharmacol 60: S1–S14. https://doi.org/10.1016/j.yrtph.2011.03.010 <https://www.ncbi.nlm.nih.gov/pubmed/21466831>
13. OECD (2003) Descriptions of selected generic key terms used in chemical hazard/Risk assessment. OECD Series on Testing and Assessment. Number 44. Joint Project with IPCS on the Harmonisation of Hazard/Risk Assessment Terminology. OECD Environment Directorate, Environment, Health and Safety Division, Paris, France
14. US EPA (2002) Framework for Cumulative Risk Assessment. Washington, DC, US Environmental Protection Agency (EPA/630/P-02/001A).
15. US-EPA (2011) Exposure Factors Handbook, Glossary of terms. EPA/600/P-95/002Fa-c). Washington, DC: US Environmental Protection Agency. [EPA Report] HERO ID: 594981 <https://hero.epa.gov/hero/index.cfm/project/page/page/1/rows/10/sort/year%20desc/format/list/project_id/1854/usage_searchType/any/usage_id/8068>
16. Van Leeuwen, C., Vermeire, T.G. eds. (2007) Risk assessment of chemicals: an introduction, second edition. Springer, Dordrecht, The Netherlands.
17. WHO/IPCS (2004) PCS risk assessment terminology-- (IPCS harmonization project ; document no. 1) part 1, IPCS/OECD key generic terms used in chemical hazard/risk assessment / International Programme on Chemical Safety Joint Project with OECD on the Harmonization of Hazard/Risk Assessment Terminology, part -2, IPCS glossary of key exposure assessment terminology / IPCS project on the Harmonisation of Approaches to the Assessment of Risk from Exposure to Chemicals. ISBN 92 4 156267 6 (LC/NLM classification: QV 15) World Health Organization 2004
18. WHO/IPCS (2008) Uncertainty and data quality in exposure assessment. IPCS harmonization project document; no. 6. Part 1: guidance document on characterizing and communicating uncertainty in exposure assessment. Part 2: hallmarks of data quality in chemical exposure assessment. International Programme on Chemical Safety. II.Series. ISBN 978 92 4 156376 5 (NLM classification: QT 140)
19. WHO/IPCS (2009) Assessment of combined exposures to multiple chemicals: report of a WHO/IPCS international workshop on aggregate/cumulative risk assessment. IPCS harmonization project document; no. 7.
20. Wild CP (2005) Complementing the genome with an "exposome": the outstanding challenge of environmental exposure measurement in molecular epidemiology. Cancer Epidemiol Biomarkers Prev 2005;14:1847–50

1. Definitions made by the ISES expert group are not referenced. The reference is this paper. [↑](#footnote-ref-1)
